# An unexpected extraluminal foreign body

**DOI:** 10.1055/a-2774-3445

**Published:** 2026-03-26

**Authors:** Xiaojing Du, AiPing Xu, Meidong Xu

**Affiliations:** 1 66324 Endoscopy Center, Department of Gastroenterology, Shanghai East Hospital, School of Medicine, Tongji University, Shanghai, China

**Fig. 1:**
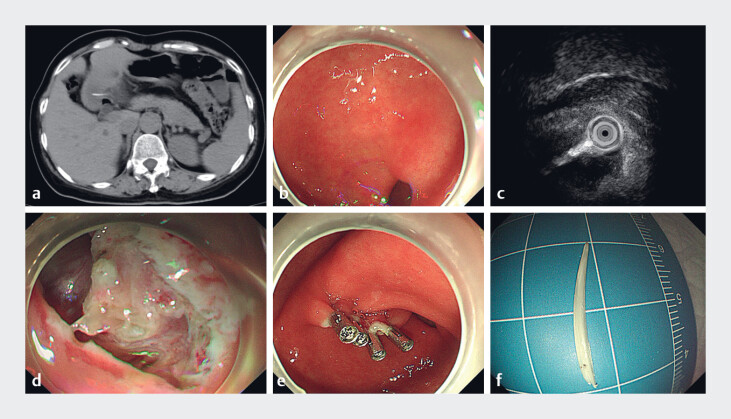
**a** A CT scan showed a dense shadow penetrating the gastric wall. **b** Endoscopic white light manifestation. **c** EUS showed a bright line extending from the cavity. **d** A foreign body wrapped in the inflammatory granulation tissue. **e** The wound after removing a foreign body with endoscopy clips. **f** Removed the fishbone. CT, computed tomography; EUS, endoscopic ultrasonography.

A 55-year-old female breast cancer patient underwent routine follow-up abdominal computed tomography, which revealed a dense shadow in the gastric antrum that had penetrated the gastric wall ( Fig. 1 **a** ). She has no discomfort symptoms. Routine blood tests upon admission showed normal levels of inflammatory markers, including white blood cells, C-reactive protein, and procalcitonin. She received endoscopic treatment in our center. We found a cord-like submucosal protrusion with a smooth surface in the antrum of the stomach ( Fig. 1 **b** ). Mini-probe endoscopic ultrasonography (EUS) detected a hyperechoic linear structure extending outward from the cavity ( Fig. 1 **c** ).

Given that foreign bodies may lead to serious complications 1 , endoscopic treatment was performed ( Video 1 ). After layer-by-layer dissection along the raised area to the muscularis propria, a translucent, needle-like foreign body was exposed at one end, surrounded by inflammatory granulation tissues ( Fig. 1 **d** ). Attempts to remove the foreign body with foreign forceps were unsuccessful. Subsequently, further dissection and compression of the surrounding tissues were performed, ultimately successfully extracting the foreign body, and the wound was closed with endoscopic clips ( Fig. 1 **e** ). After removal, the foreign body was identified as a fishbone measuring approximately 2.0 cm ( Fig. 1 **f** ).

Endoscopic therapy for an extraluminal foreign body.Video 1

The patient had an uneventful recovery, initiated a clear liquid diet on postoperative day 1, and was discharged on day 2.

Extraluminal migration of the fishbone in the gaster is a rare complication, and cases where the fishbone cannot be visualized endoscopically are even rarer. Here, EUS localization and endoscopic submucosal dissection were combined to remove the foreign body, providing an effective therapeutic paradigm for endoscopic extracorporeal foreign body extraction.

Endoscopy_UCTN_Code_TTT_1AO_2AB
